# Supplementary material for: Genomic landscape of extraordinary responses in metastatic breast cancer
Source: Commun Biol. 2021 Apr 9;4:449. doi: 10.1038/s42003-021-01973-x (PMC8035393; doi:10.1038/s42003-021-01973-x)
Supplement: Supplementary file 2 — Supplementary Information [file 42003_2021_1973_MOESM2_ESM.pdf]

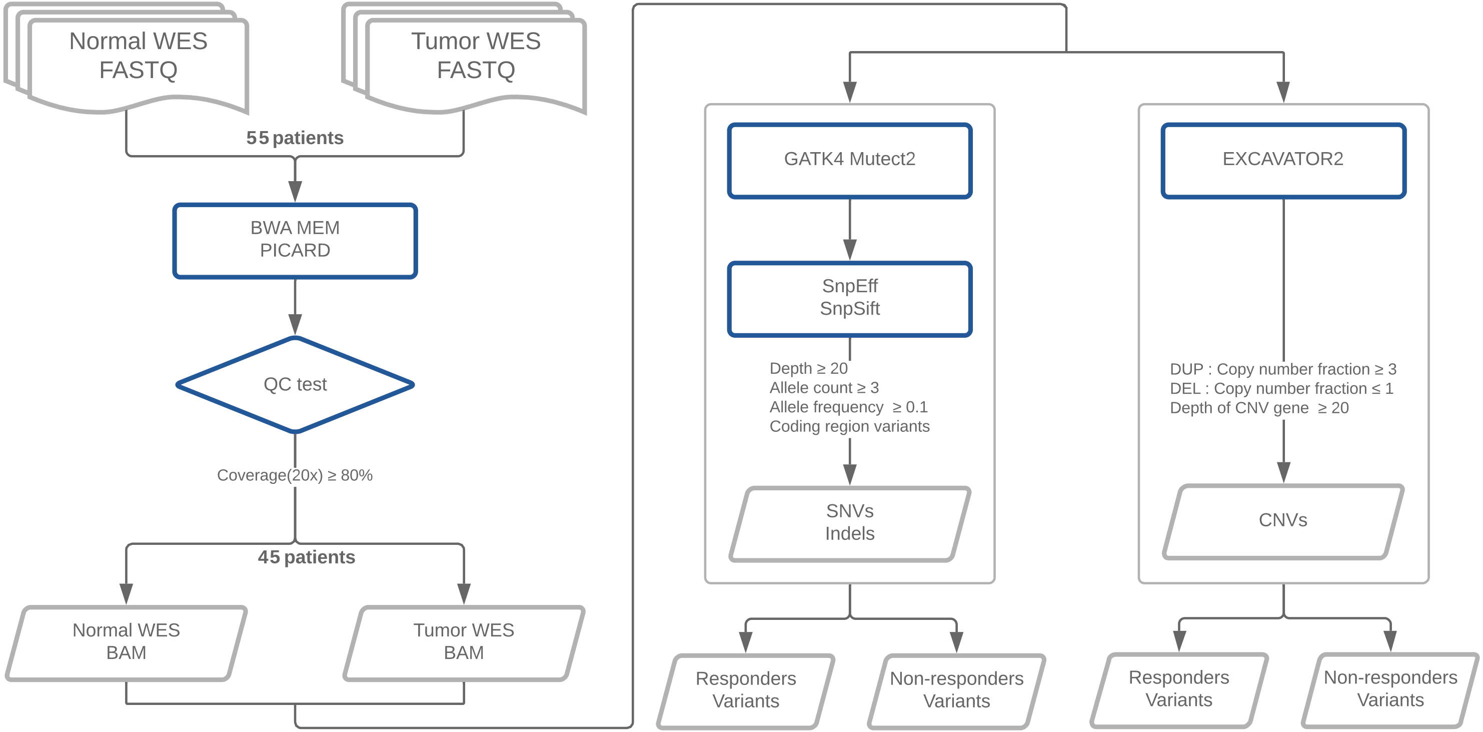

Supplementary Figure 1 Flow chart of genome variant analysis

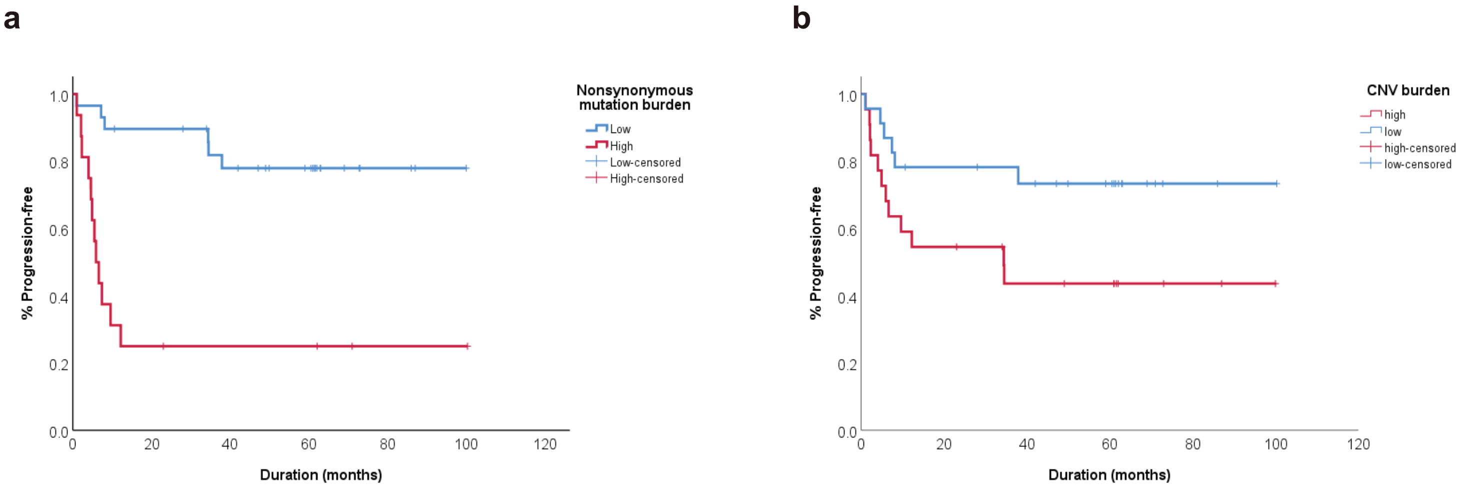

Supplementary Figure 2 (a) Kaplan-Meirer curve of progression-free survival (PFS) comparing high nonsynonymous mutational burden vs. low nonsynonymous mutational burden. The median PFS of nonsynonymous mutational burden was 5.9 months (95% CI, 3.61~8.18),
and the median PFS of low nonsynonymous mutational burden was not reached (P<0.001 by log-rank).
(b) Kaplan-Meier curve of progression-free survival (PFS) comparing high CNV burden vs. low CNV burden.
The median PFS of high CNV burden was 34.4 months (95% CI, 0-69.38),
and the median PFS of low CNV burden was not reached (P=0.032 by log-rank).

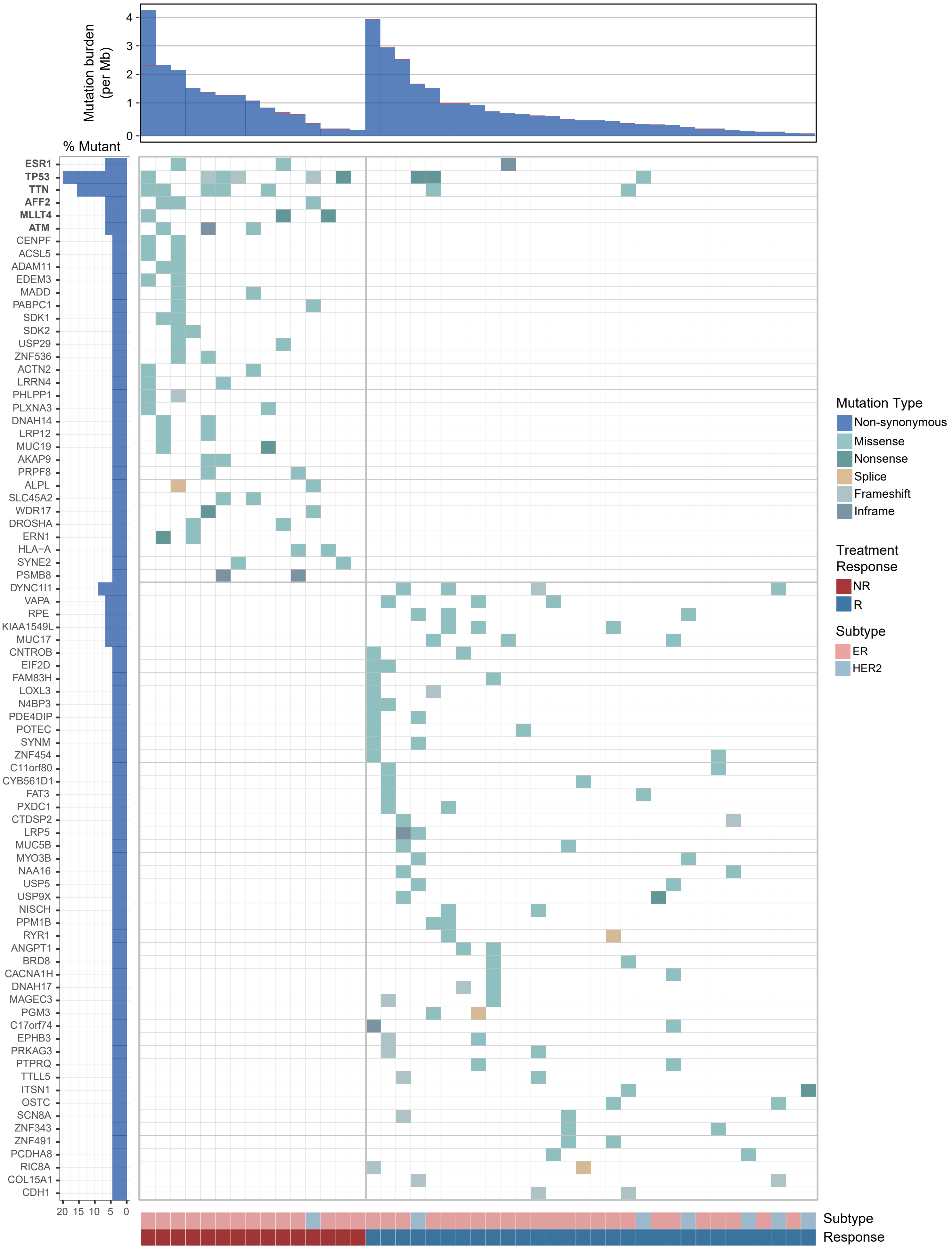

Supplementary Figure 3 Mutational landscape of group-specific and recurrently mutated genes between the responders and non-responders. The top six genes are specially added on top of the mutational landscape: five non-responder-specific genes (AFF2, TTN, TP53, ATM, and MLLT4) and ERS1 is associated with resistance to aromatase inhibitors. Out of a total of 1662 mutated genes, 32 were non-responder-specific and 79 were responder-specific.

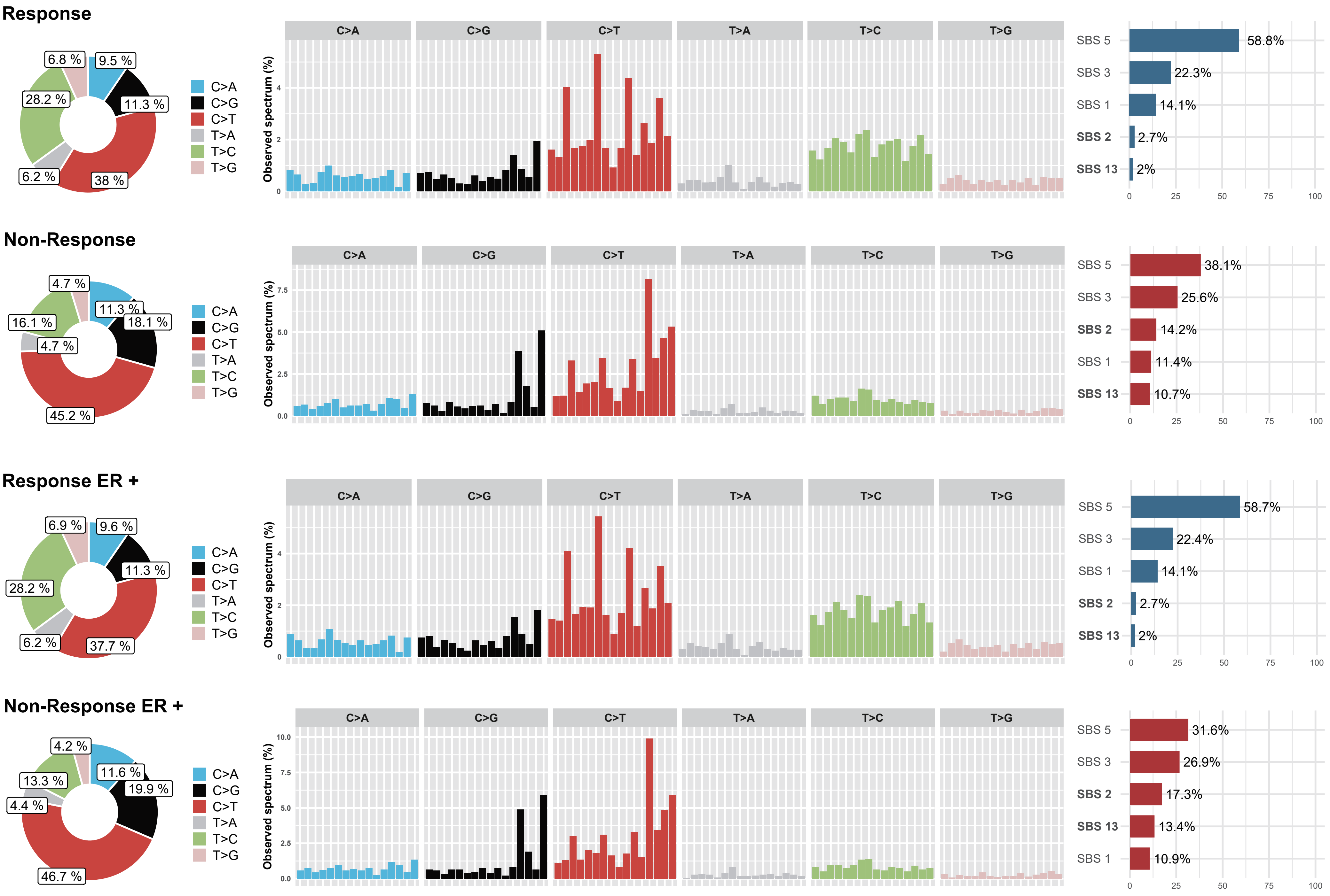

Supplementary Figure 4 Comparison of COSMIC signatures between the responders and non-responders. COSMIC signature analysis was also conducted on ER-positive patients.

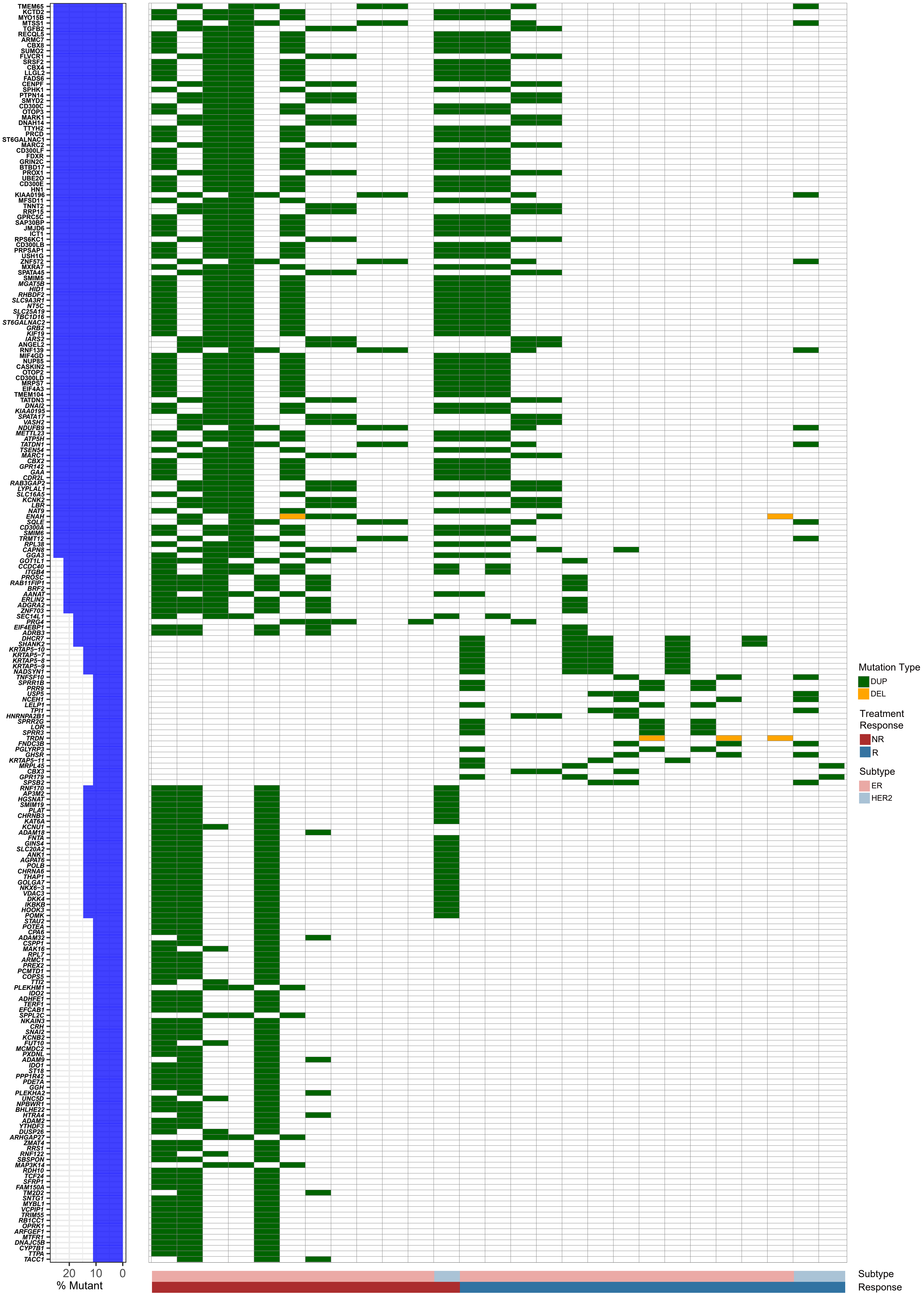

Supplementary Figure 5 The oncoplot of copy number variants in the cohort.
