# Supplementary material for: Genomic landscape of extraordinary responses in metastatic breast cancer
Source: Commun Biol. 2021 Apr 9;4:449. doi: 10.1038/s42003-021-01973-x (PMC8035393; doi:10.1038/s42003-021-01973-x)
Supplement: Supplementary file 3 — Description of Supplementary Files [file 42003_2021_1973_MOESM3_ESM.pdf]

Description of Additional Supplementary Files

File Name: Supplementary Data 1

Description: Raw data for Figures 2 to 5.
